# Supplementary material for: Structure of engineered hepatitis C virus E1E2 ectodomain in complex with neutralizing antibodies
Source: Nat Commun. 2023 Jul 5;14:3980. doi: 10.1038/s41467-023-39659-z (PMC10322937; doi:10.1038/s41467-023-39659-z)
Supplement: Supplementary file 1 — Supplementary Information [file 41467_2023_39659_MOESM1_ESM.pdf]

SUPPLEMENTARY INFORMATION

Structure of engineered hepatitis C virus E1E2 ectodomain in complex with neutralizing antibodies

Metcalf, M.C.[1,2], Janus, B.M.[1,2], Yin, R.[1,2], Wang, R.[2], Guest, J.D. [1,2], Pozharski, E.[2,3,4], Law, M.[5], Mariuzza, R.A.[1,2], Toth, E.A.[2], Pierce, B.G. [1,2], Fuerst, T.R.[1,2], Ofek, G.[1,2,#]

[1]Department of Cell Biology and Molecular Genetics, University of Maryland, College Park, MD, United States

[2]Institute for Bioscience and Biotechnology Research, University of Maryland, Rockville, MD, United States

[3]Center for Biomolecular Therapeutics, University of Maryland School of Medicine, Baltimore, MD, United States

[4]Department of Biochemistry and Molecular Biology, University of Maryland School of Medicine, Baltimore, MD, United States

[5]Department of Immunology and Microbiology, The Scripps Research Institute, La Jolla, CA, United States

[#]Correspondence: Gilad Ofek, gofek@umd.edu

This PDF file includes:
Supplementary Figures 1-8
Supplementary Table 1

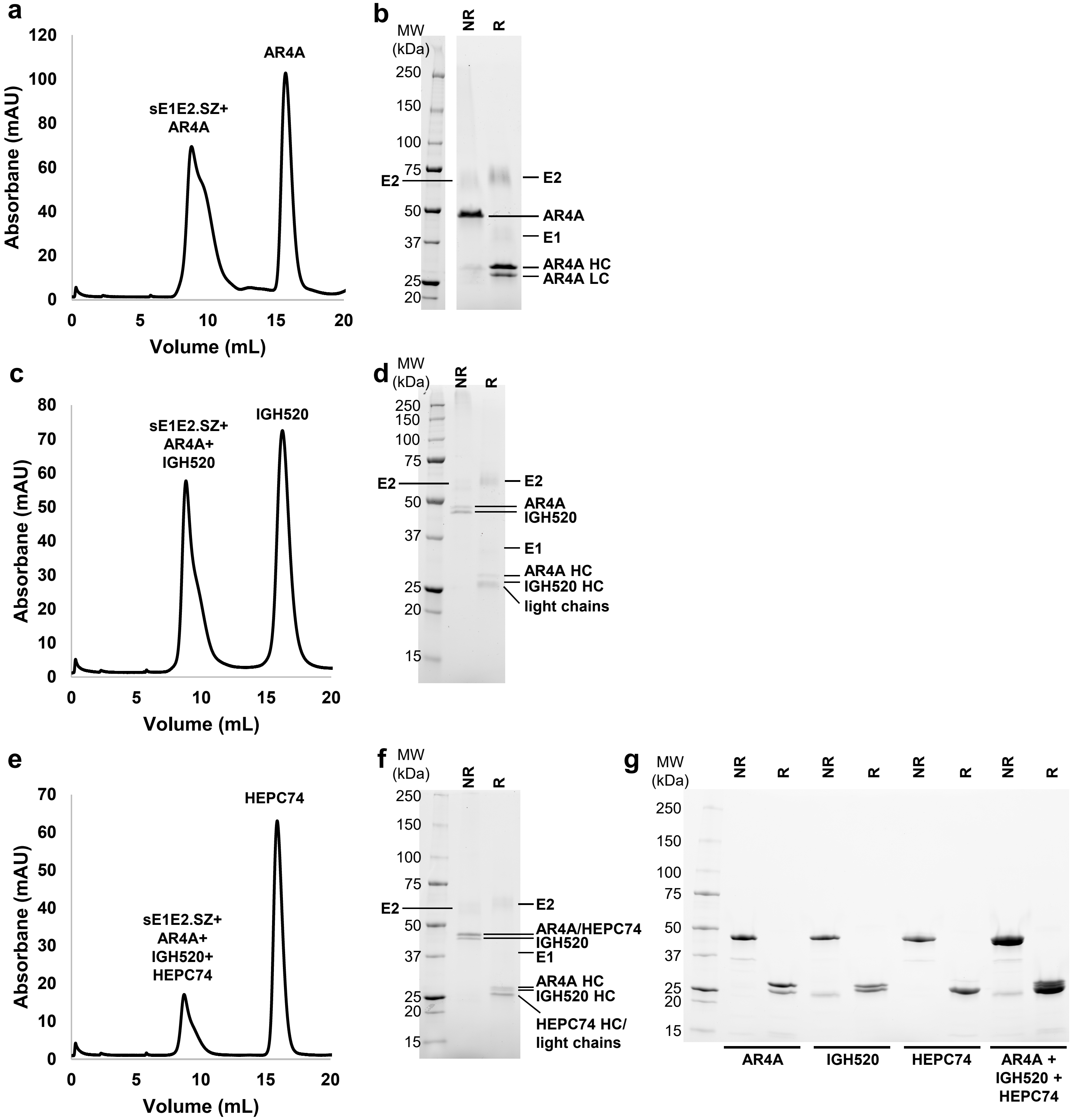

Supplementary Fig. 1. Preparation of sE1E2.SZ quinary complex. (a) Size-exclusion chromatogram of sE1E2.SZ-AR4A Fab ternary complex, left peak, with unbound AR4A Fab eluting in the right peak. **(b)** Stain-free SDS-PAGE of the sE1E2.SZ-AR4A complex from **a**, under reducing (R) and non-reducing (NR) conditions. **(c)** Size-exclusion chromatogram of sE1E2.SZ-AR4A-IGH520 quaternary complex, left peak, with unbound IGH520 Fab eluting in the right peak. **(d)** Stain-free SDS-PAGE of the sE1E2.SZ-AR4A-IGH520 complex under reducing (R) and non-reducing (NR) conditions. **(e)** Size-exclusion chromatogram of sE1E2.SZ-AR4A-IGH520-HEPC74 quinary complex, left peak, with unbound HEPC74 Fab eluting in the right peak. **(f)** Stain-free SDS-PAGE of sE1E2.SZ-AR4A-IGH520-HEPC74 complex under reducing (R) and non-reducing (NR) conditions. **(g)** Stain-free gel of AR4A, IGH520, and HEPC74 Fabs alone, and of a molar equivalent combination of the three under reducing and non-reducing conditions.

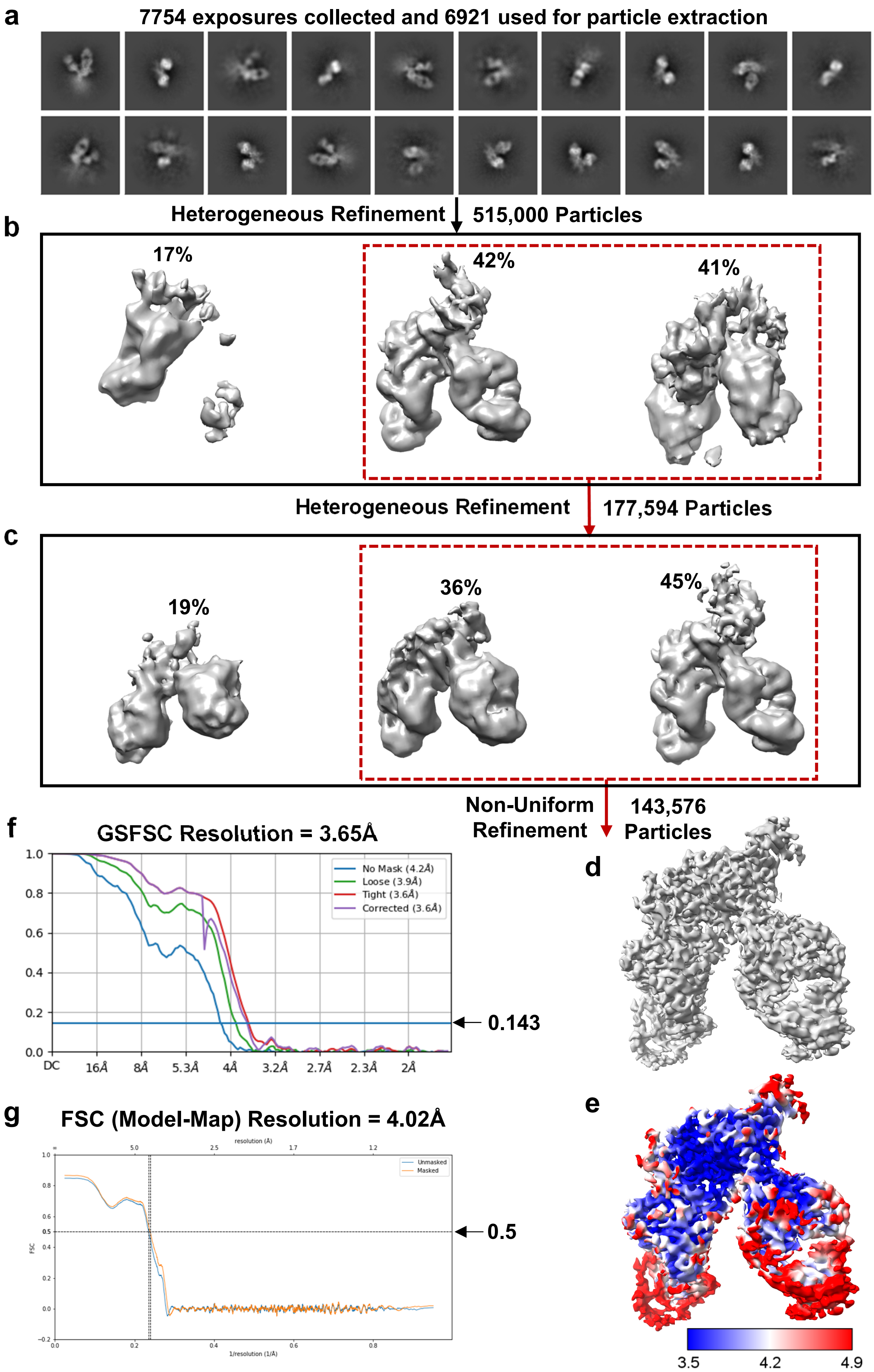

Supplementary Fig. 2. cryo-EM pipeline for primary map. (**a**) Particles picked using the cryoSPARC template picking program were used for 2D classification from which 515,000 particles that were selected for further processing. (**b**) *Ab initio* reconstructions (n=3) using the particles selected from 2D classification were performed followed by heterogenous refinement of the three classes. Two of the resulting classes were pooled for further refinement. (**c**) An additional round of *ab initio* reconstruction (n=3) using the particles selected from (**b**) was performed followed by heterogenous refinement. Two of the classes were pooled and further refined using non-uniform refinement. The map resulting from non-uniform refinement shown in gray (**d**) and colored by local resolution estimate as calculated using the local resolution tool in cryoSPARC (**e**). Gold-standard fourier shell correlation (GSFSC) curve using a threshold of 0.143 (**f**), and map-model fourier shell correlation curve using a threshold of 0.5 (**g**).

Supplementary Table 1. CryoEM data collection, processing, and refinement

	sE1E2.SZ+AR4A+HEPC74+IGH520 (EMDB-29419) (PDB 8FSJ)
**Data collection and processing**	
Magnification (nominal)	45,000
Voltage (kV)	200
Electron exposure (e–/Å^2)	47
Defocus range (μm)	0.5-2.5
Pixel size (Å)	0.889
Symmetry imposed	C1
Initial particle images (no.)	2,620,845
Final particle images (no.)	143,576
Map resolution (Å)	3.65
FSC threshold	0.143
Map resolution range (Å)	3.0-8.0
**Refinement**	
Map-Model FSC (Å)	4.02
FSC threshold	0.5
Map sharpening *B* factor (Å^2)	114
Model composition	
Non-hydrogen atoms	9855
Protein residues	1257
Ligands	28
B factors (Å^2)	
Protein (mean)	65.34
Ligand (mean)	81.00
R.m.s. deviations	
Bond lengths (Å)	0.003
Bond angles (°)	0.590
Validation	
MolProbity score	1.84
Clashscore	7.75
Poor rotamers (%)	0
Ramachandran plot	
Favored (%)	93.7
Allowed (%)	6.3
Disallowed (%)	0

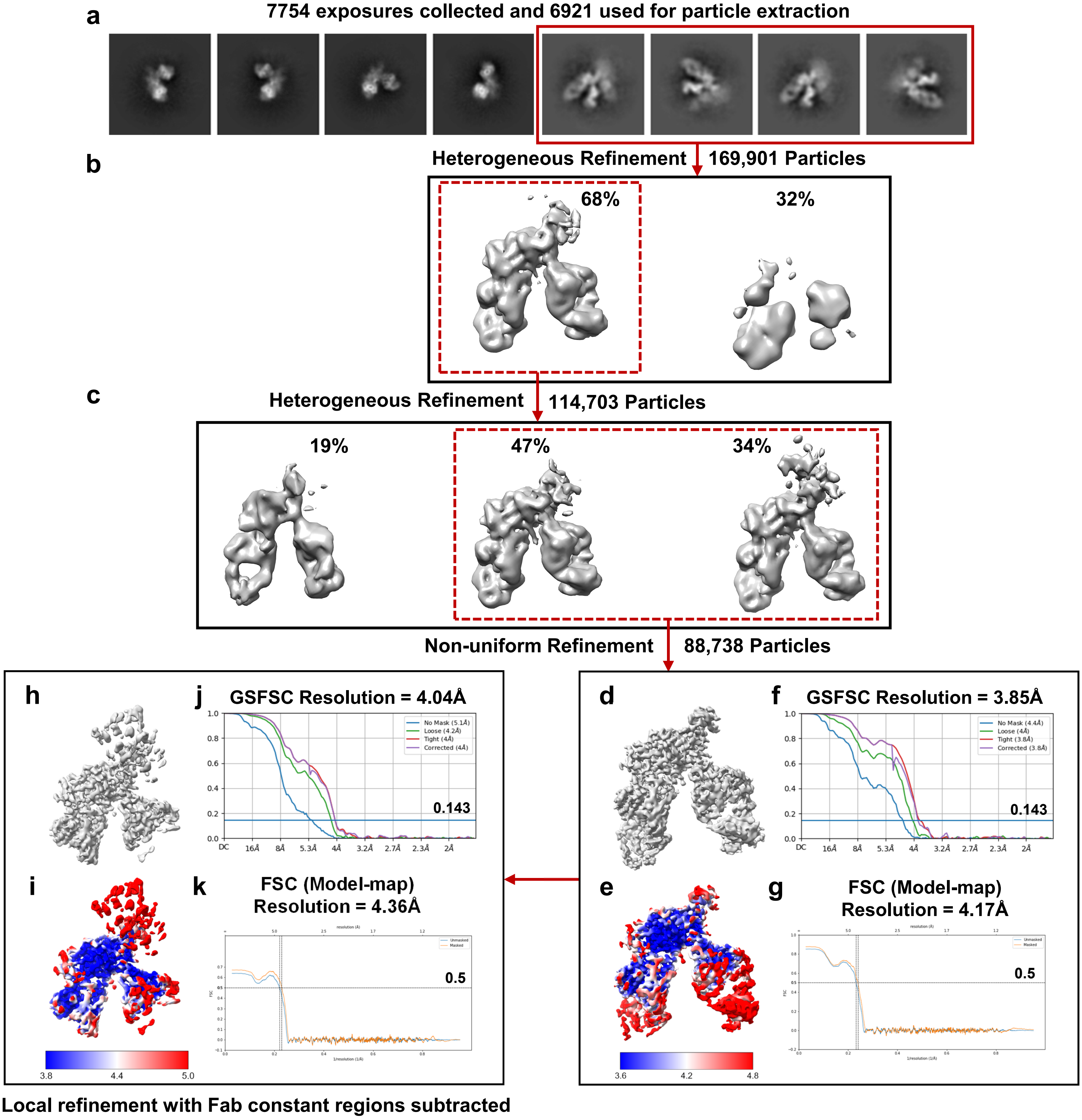

Supplementary Fig. 3. cryo-EM pipeline for extended E1. (**a**) Shown boxed are representative 2D classes from 13 classes with visible IGH520 signal. (**b**) *Ab initio* reconstructions (n=2) using the particles from (**a**) were performed followed by heterogenous refinement. One class was selected for further refinement. (**c**) Particles from (**b**) were then applied to an additional round of *ab initio* reconstruction (n=3) and heterogeneous refinement. The classes with the most volume corresponding to IGH520 were selected for non-uniform refinement. (**d**) The map resulting from non-uniform refinement shown in gray and colored by local resolution estimate as calculated using the local resolution tool in cryoSPARC (**e**). Gold-standard fourier shell correlation (GSFSC) curve using a threshold of 0.143 (**f**), and map-model fourier shell correlation curve using a threshold of 0.5 (**g**). Particle subtraction masking the Fc regions of HEPC74 and AR4A was performed on the particle stack and local refinement was performed. (**h**) The map resulting from local refinement shown in gray and colored by local resolution estimate (**i**). Gold-standard fourier shell correlation (GSFSC) curve using a threshold of 0.143 (**j**), and map-model fourier shell correlation curve using a threshold of 0.5 (**k**).

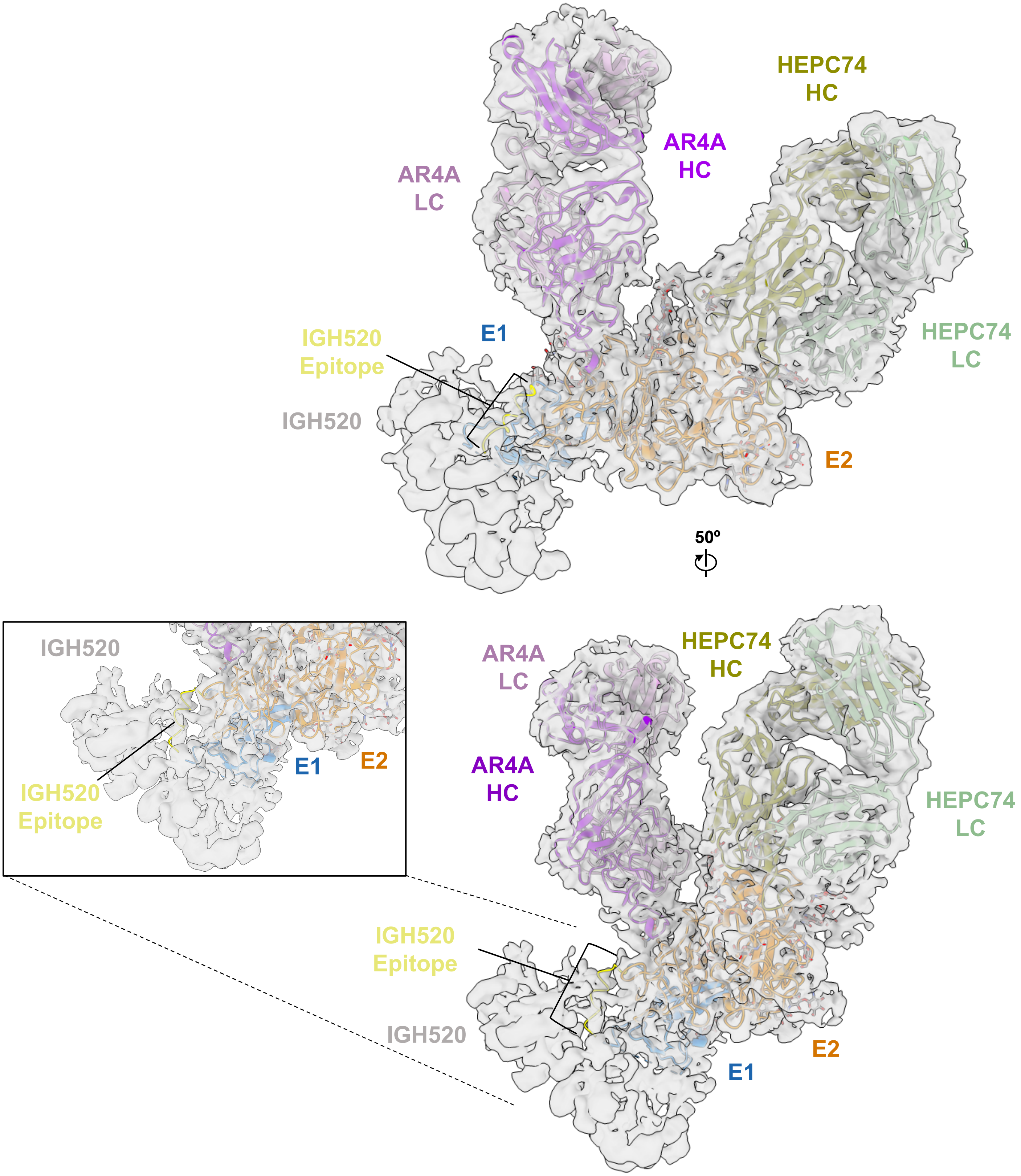

Supplementary Fig. 4. Merged composite Cryo-EM map. A cryo-EM map with extended density for IGH520 and E1 was generated by merging the local resolution map from **Supplementary Fig. 3** and the primary non-uniform map from **Supplementary Fig. 2**. The merged cryo-EM map is shown as a semi-transparent surface representation (gray) with the structural model of E1E2 bound antibodies AR4A and HEPC74 colored as in **Fig. 1**. The putative IGH520 epitope in E1 is colored yellow. HC, heavy chain; LC, light chain.

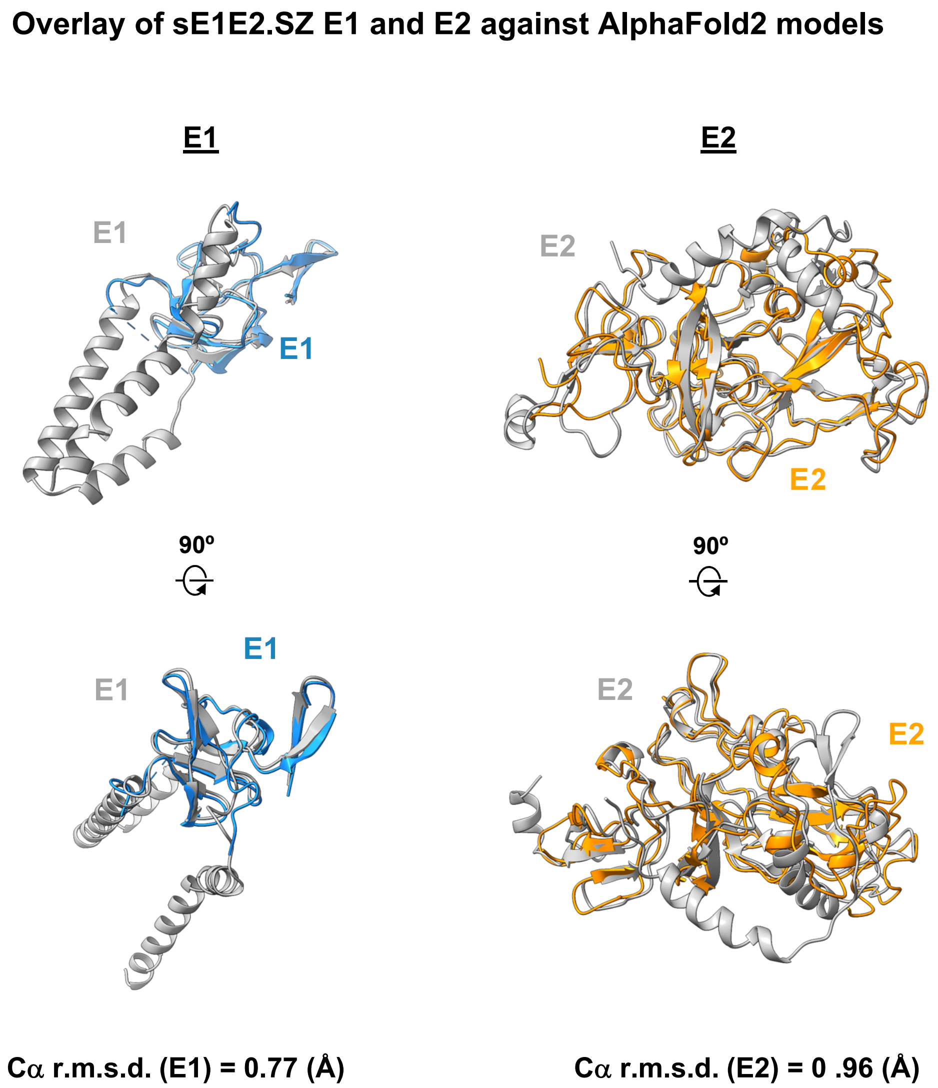

Supplementary Fig. 5. Structural comparison of individual E1 and E2 subunits from sE1E2.SZ structure and AlphaFold2-generated models. Structural alignment of the individual E1 (blue) and E2 (orange) subunits of sE1E2.SZ against respective AlphaFold2-predicted models of each (gray).

Overlay of HEPC74-bound sE1E2.SZ E2 vs. HEPC74-bound sE2 (6MEH)

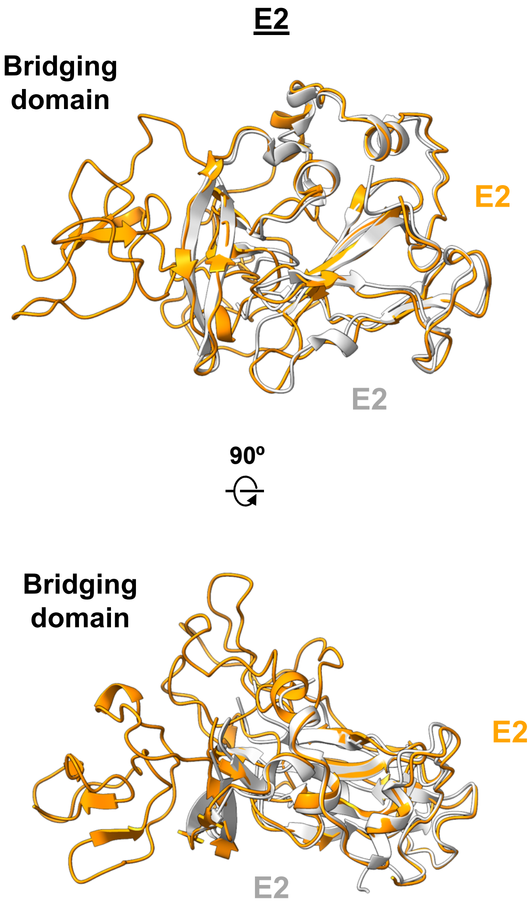

Cα r.m.s.d. = 1.02 (Å)

Supplementary Fig. 6. Structural comparison of HEPC74-bound sE1E2.SZ E2 vs. HEPC74-bound sE2. The E2 subunit of the sE1E2.SZ (orange) was aligned against the previously reported HEPC74-bound E2 crystal structure (PDB ID 6MEH, gray) using common residues between them.

Overlay of sE1E2.SZ E2 against CD81-bound sE2 (7MWX)

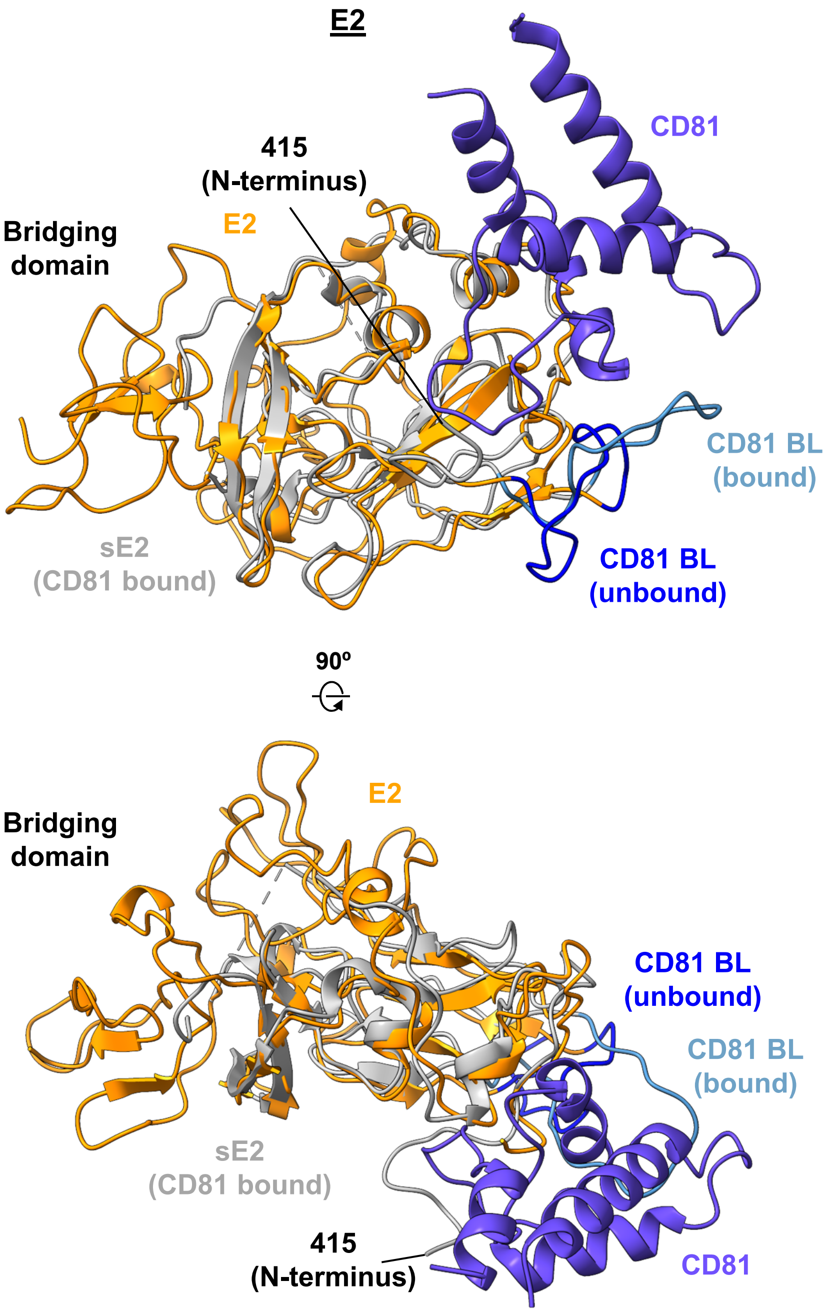

Cα r.m.s.d. (E2) = 0.97 (Å)

Supplementary Fig. 7. Structural comparison of sE1E2.SZ E2 against CD81-bound E2. E2 subunit of sE1E2.SZ (orange) overlaid with CD81-bound E2 structure (PDB ID 7MWX, gray). CD81 is depicted in purple. CD81 binding loops of the sE1E2.SZ and 7MWX structures are shown in blue and light blue, respectively.

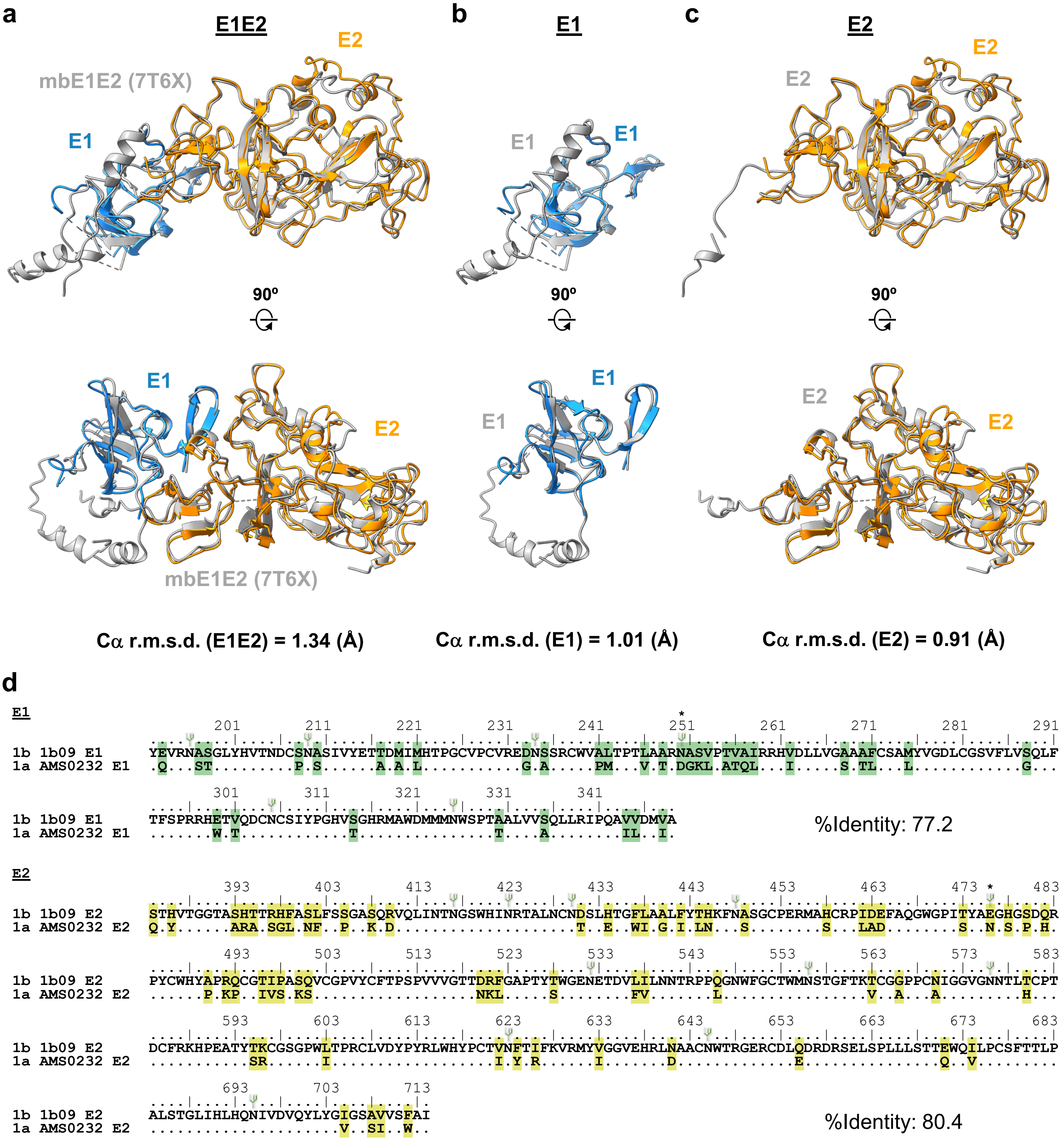

Supplementary Fig. 8. Structural and sequence comparison of genotype 1b sE1E2.SZ vs. genotype 1a membrane-extracted E1E2. (a) sE1E2.SZ heterodimer structure (colored as in **Fig. 1**) overlaid against the membrane-extracted E1E2 heterodimer structure (PDB 7T6X, gray), shown in 90º orientations. **(b)** Superposition of individual E1 subunits in sE1E2.SZ (blue) and membrane-extracted E1E2 (gray), shown in 90º orientations. **(c)** Superposition of individual E2 subunits in sE1E2.SZ (orange) and membrane-extracted E1E2 structure (gray), shown in 90º orientations. **(d)** Amino acid sequence alignment of E1 and E2 ectodomains of sE1E2.SZ genotype 1b (1b09) and membrane-extracted E1E2 genotype 1a (AMS0232). Residue positions that diverged in sequence are shaded green for E1 and yellow for E2. Transmembrane domains were excluded from the alignments. N-linked glycans are annotated on the sequence. *, N-linked glycan sequons that diverged between the two genotypes.
