# Supplementary material for: Structure of engineered hepatitis C virus E1E2 ectodomain in complex with neutralizing antibodies
Source: Nat Commun. 2023 Jul 5;14:3980. doi: 10.1038/s41467-023-39659-z (PMC10322937; doi:10.1038/s41467-023-39659-z)
Supplement: Supplementary file 3 — Reporting Summary [file 41467_2023_39659_MOESM3_ESM.pdf]

nature portfolio

Corresponding author(s): Gilad Ofek

Last updated by author(s): Jun 4, 2023

Reporting Summary

Nature Portfolio wishes to improve the reproducibility of the work that we publish. This form provides structure for consistency and transparency in reporting. For further information on Nature Portfolio policies, see our Editorial Policies and the Editorial Policy Checklist.

Statistics

For all statistical analyses, confirm that the following items are present in the figure legend, table legend, main text, or Methods section.

n/a	Confirmed	
☒	☐	The exact sample size (*n*) for each experimental group/condition, given as a discrete number and unit of measurement
☒	☐	A statement on whether measurements were taken from distinct samples or whether the same sample was measured repeatedly
☒	☐	The statistical test(s) used AND whether they are one- or two-sided<br>*Only common tests should be described solely by name; describe more complex techniques in the Methods section.*
☒	☐	A description of all covariates tested
☒	☐	A description of any assumptions or corrections, such as tests of normality and adjustment for multiple comparisons
☒	☐	A full description of the statistical parameters including central tendency (e.g. means) or other basic estimates (e.g. regression coefficient) AND variation (e.g. standard deviation) or associated estimates of uncertainty (e.g. confidence intervals)
☒	☐	For null hypothesis testing, the test statistic (e.g. *F*, *t*, *r*) with confidence intervals, effect sizes, degrees of freedom and *P* value noted<br>*Give P values as exact values whenever suitable.*
☒	☐	For Bayesian analysis, information on the choice of priors and Markov chain Monte Carlo settings
☒	☐	For hierarchical and complex designs, identification of the appropriate level for tests and full reporting of outcomes
☒	☐	Estimates of effect sizes (e.g. Cohen's *d*, Pearson's *r*), indicating how they were calculated

Our web collection on statistics for biologists contains articles on many of the points above.

Software and code

Policy information about availability of computer code

Data collection	Cryo-EM data was collected using SerialEM v3.8 data collection software.
Data analysis	CryoSPARC v4.2.1, Phenix v1.20.1-4487, Molprobity v4.5.2, UCSF ChimeraX v1.4, UCSF Chimera v1.15, PISA v1.48, and CCP4i2 v7.1.018 were used for data processing and analysis.

For manuscripts utilizing custom algorithms or software that are central to the research but not yet described in published literature, software must be made available to editors and reviewers. We strongly encourage code deposition in a community repository (e.g. GitHub). See the Nature Portfolio guidelines for submitting code & software for further information.

Data

Policy information about availability of data

All manuscripts must include a data availability statement. This statement should provide the following information, where applicable:

- Accession codes, unique identifiers, or web links for publicly available datasets
- A description of any restrictions on data availability
- For clinical datasets or third party data, please ensure that the statement adheres to our policy

The cryo-EM data generated in this study have been deposited in the Electron Microscopy Data Bank under accession code EMD-29419 [https://www.ebi.ac.uk/pdbe/entry/emdb/EMD-29419]. The atomic model generated in this study has been deposited in the Protein Data Bank under accession code 8FSJ [http://doi.org/10.2210/pdb8FSJ/pdb]. All other data supporting the findings of this study are available with the Article and the Supplementary Information. Source data

are provided with this paper as a Source Data file. Previously published structures used in the design of sE1E2.SZ are available under PDB accession codes 3HE5 [http://doi.org/10.2210/pdb3HE5/pdb] and 1FOS [http://doi.org/10.2210/pdb1FOS /pdb]. Previously published structures used for comparative analyses are available under PDB accession codes 7T6X [https://doi.org/10.2210/pdb7t6x/pdb], 4UOI [https://doi.org/10.2210/pdb4uoi/pdb], 6MEH [http://doi.org/10.2210/pdb6MEH/pdb], 6UYG [https://doi.org/10.2210/pdb6uyg/pdb], 6W03 [https://doi.org/10.2210/pdb6w03/pdb], 7MWX [https://doi.org/10.2210/pdb7mwx/pdb], 6MEJ [https://doi.org/10.2210/pdb6mej/pdb], 6BKB [https://doi.org/10.2210/pdb6bkb/pdb], 6UYF [https://doi.org/10.2210/pdb6uyf/pdb], 6URH [https://doi.org/10.2210/pdb6urh/pdb], 6UYM [https://doi.org/10.2210/pdb6uym/pdb], 6BKD [https://doi.org/10.2210/pdb6bkd/pdb], 7JTG [https://doi.org/10.2210/pdb7jtg/pdb], 7JTF [https://doi.org/10.2210/pdb7jtf/pdb], 6WO4 [https://doi.org/10.2210/pdb6wo4/pdb], and 7RFC [https://doi.org/10.2210/pdb7rfc/pdb].

Research involving human participants, their data, or biological material

Policy information about studies with human participants or human data. See also policy information about sex, gender (identity/presentation), and sexual orientation and race, ethnicity and racism.

Reporting on sex and gender	n/a
Reporting on race, ethnicity, or other socially relevant groupings	n/a
Population characteristics	n/a
Recruitment	n/a
Ethics oversight	n/a

Note that full information on the approval of the study protocol must also be provided in the manuscript.

Field-specific reporting

Please select the one below that is the best fit for your research. If you are not sure, read the appropriate sections before making your selection.

☒ Life sciences ☐ Behavioural & social sciences ☐ Ecological, evolutionary & environmental sciences

For a reference copy of the document with all sections, see nature.com/documents/nr-reporting-summary-flat.pdf

Life sciences study design

All studies must disclose on these points even when the disclosure is negative.

Sample size	7,754 micrographs were collected in total. Of these, 6,921 micrographs were selected after motion correction and ctf estimation, using a ctf resolution estimate cut-off of 4Å. After multiple rounds of 2D and 3D class sorting a stack of 143,576 particles was used to generate the cryo-EM maps used in this study.
Data exclusions	Particles that were not of the 2D/3D classes used in the final 3D reconstruction were excluded from this dataset.
Replication	The estimated resolution was determined by the Fourier shell correlation between two half-maps using a threshold of 0.143, establishing the resolution, which is reproducible from the processed data.
Randomization	The dataset was randomly split into two halves and refined independently in cryoSPARC. The estimated resolution, using a Fourier shell correlation threshold of 0.143, was determined using the two half-maps.
Blinding	Blinding was not a relevant feature in this study. Particle assignment between the half-sets was performed randomly by cryoSPARC.

Reporting for specific materials, systems and methods

We require information from authors about some types of materials, experimental systems and methods used in many studies. Here, indicate whether each material, system or method listed is relevant to your study. If you are not sure if a list item applies to your research, read the appropriate section before selecting a response.

Materials & experimental systems

n/a	Involved in the study
☐	☒ Antibodies
☐	☒ Eukaryotic cell lines
☒	☐ Palaeontology and archaeology
☒	☐ Animals and other organisms
☒	☐ Clinical data
☒	☐ Dual use research of concern
☒	☐ Plants

Methods

n/a	Involved in the study
☒	☐ ChIP-seq
☒	☐ Flow cytometry
☒	☐ MRI-based neuroimaging

Antibodies

Antibodies used	Heavy and light chains for the antigen binding fragments of antibodies HEPC74, AR4A, and IGH520 were generated by gene synthesis and recombinantly expressed. Antibody sequences were deposited in the PDB deposition.
Validation	Antibody validation was accomplished through biochemical analysis, functional analysis, and structural characterization, as presented in the submitted manuscript.

Eukaryotic cell lines

Policy information about cell lines and Sex and Gender in Research

Cell line source(s)	Commercially available Expi293F cells or Expi293F GnTI- Cells were acquired from Thermo Fisher Scientific (catalog #s A14527 and #A39240).
Authentication	No authentication was perfomed.
Mycoplasma contamination	Cells were not tested for mycoplasma contamination.
Commonly misidentified lines (See ICLAC register)	No commonly misidentified cell lines were used in the study.
